# Evaluation of Gait Parameters on Subjects with Hallux Limitus Using an Optogait Sensor System: A Case–Control Study

**DOI:** 10.3390/medicina59091519

**Published:** 2023-08-23

**Authors:** Aurora Castro-Méndez, Francisco Javier Canca-Sánchez, Manuel Pabón-Carrasco, Ana María Jiménez-Cebrián, Antonio Córdoba-Fernández

**Affiliations:** 1 Podiatry Department, Universidad de Sevilla, Avicena St., 41009 Sevilla, Spain; mpabon2@us.es; 2 Podiatry Department, Universidad de Málaga, Arquitecto Francisco Peñalosa, 3, 29071 Málaga, Spain; fjcanca@uma.es; 3 Instituto de Investigación Biomédica de Málaga (IBIMA), Universidad de Málaga, Arquitecto Francisco Peñalosa, 3, 29071 Málaga, Spain; amjimenezc@uma.es

**Keywords:** hallux limitus, gait cycle, imbalances, Optogait

## Abstract

*Background and Objetives*: The foot is a part of the body’s kinetic chain and needs to be efficient during the entire gait cycle. Electronic Sensor Gait analysis is useful and an important tool within the area of podiatry to assess the physical state of patients that helps the comprehensive intervention in situations where the daily activity is limited. The aim of this research is to evaluate if the presence of a hallux limitus (HL) can alter gait space–time parameters and consequently can affect the take-off phase of the gait and the limitation of the range of motion (ROM) of the hallux. *Materials and Methods*: A case–control study was designed to verify whether there are alterations in the spatiotemporal parameters of the gait cycle between subjects with structural HL compared to the group of subjects with a normal hallux range. A total of *n* = 138 participants, cases (68 HL subjects) and healthy controls (70 subjects) were studied using an OptoGait LED sensor system to identify gait imbalances using OptoGait photocell gait analysis sensors. *Results*: Significant differences were found between the two groups with respect to stride length, gait cycle duration in seconds (for both feet) and for total stride and load response (*p* < 0.05). *Conclusions*: The limitation of the Hallux ROM may alter the normal gait patterns measured with an Optogait system. The early identification and treatment of gait disturbances due to HL are important to achieve normal gait physical activity to maintain a healthy lifestyle.

## 1. Introduction

Gait disturbances have been related in research to biomechanical disturbances at the foot level. The foot is one more link in the body’s kinetic chain and is considered decisive in this functional unit. It has a particularity, and its behaviour is dual during gait; it has a function as a shock-absorbing element in the contact phase or as a propellant element in the take-off phase.

During gait, the body structure is designed to pull the centre of mass to a single pivot point formed by the dorsiflexion of the first metatarsophalangeal joint (MPJ). Various studies have studied how structural and functional modifications of the MPJ can modify normal ambulation [1,2,3]. There is no scientific evidence to highlight whether the limitation of the hallux range of motion (ROM) alters spatiotemporal gait parameters. The current trend is to maintain a life as active as possible that allows healthy physical habits, which is why it is essential to take care of and prevent situations that can limit this activity.

HL is a disorder among the most common foot arthritides, with a high incidence and affects 2.5% of the population over 50 years of age. Its aetiology is associated with repeated trauma or altered biomechanic factors [4,5,6]. Structural HL is associated with a progressive and painful limitation of dorsiflexion movement and a proliferative bone response due to the appearance of osteophytes of the first MPJ [7].

It remains difficult to reach a consensus on the degree of limitation in the first MPJ dorsiflexion that should be considered HL. The minimum physiological dorsiflexion of the first MPJ required for normal gait is estimated to range from 65° to 90°. Various studies provide differing ranges for normal hallux dorsiflexion, ranging from 50° to 90°; however it is generally accepted that 65 is the minimum allowable angle for normal locomotion to occur [8,9].

The available evidence shows that cases of HL/rigidus exhibit a first metatarsal with a dorsiflexed, a plantar-flexed forefoot on the rear foot associated with pronation. It has been suggested that the function of the first MPJ may be related to the motion of the ankle joint complex, although there is controversy about it [10,11,12]. The limitation of dorsiflexion in the ankle during the stance phase in the sagittal plane may be compensated for by intrinsic dorsiflexion of the foot, but this will entail foot pronation. The eversion of the rearfoot has been theoretically linked to increased dorsiflexion of the first ray and has been described as an etiologic factor of HL [13]. The elevation of the head of the first metatarsal and the increase in tension in the plantar aponeurosis and the Achilles–calcaneal–plantar system can alter the joint dynamics in the first MPJ [10].

The effects of HL on foot kinematics have received limited attention in the literature. Some case–control studies show that the presence of structural or functional HL causes the maximum plantar pressure to build up significantly more and at a faster rate than under the first metatarsal head with the consequent increase in pressure in the lateral metatarsal heads, and the time to maximum pressure under the fourth and fifth metatarsal heads [1,3].

Gait analysis is a useful and important tool in the area of podiatry, allowing us to assess the physical state of patients with biomechanical alterations, obtaining scientific evidence that helps the comprehensive intervention of the patient. The measurement of spatiotemporal gait parameters is commonly used to assess gait in healthy and injured individuals. Several different systems are available for evaluating parameters related to walking, such as the duration of the gait cycle, walking speed, and the time taken for specific phases of walking. Due to different measurement principles, such as inertial or pressure sensors, the accurate detection of spatiotemporal events may vary between systems.

However, recent studies have shown high concordance and consistency between all existing systems in assessing basic spatiotemporal parameters [14,15]. OptoGait is a portable system of biosensors that can be mounted on a treadmill to collect spatiotemporal gait data using the more comfortable gait velocity for each participant. This device has been used for the analysis of movement and the functional assessment of patients with a normal or pathological biomechanical gait cycle, which can objectively assess the patient’s general physical conditions and identify deficiencies, postural problems, and asymmetries on the basis of data and videos. The validity and reliability of the Optoagait photoelectric cell system have been investigated in several studies [16,17].

Therefore, it is important to distinguish spatiotemporal alterations in the gait cycle with respect to HL to be compensated by podiatric intervention, which allows for the design of treatments that permit daily healthy activity. The aim of this research is to evaluate whether there are alterations in the spatiotemporal gait parameters measured by the OptoGait system in subjects with HL compared to healthy matched controls. We hypothesize that HL patients have an alteration in gait cycle parameters compared to subjects with a normal hallux movement range measured with the OptoGait system.

## 2. Materials and Methods

This research was conducted to evaluate the relationship between the spatiotemporal gait parameters evaluated with an OptoGait optical biosensor system with a treadmill programme between a group of subjects with HL (cases) and a healthy group (controls) using a cross-sectional study design.

**Figure 1:**
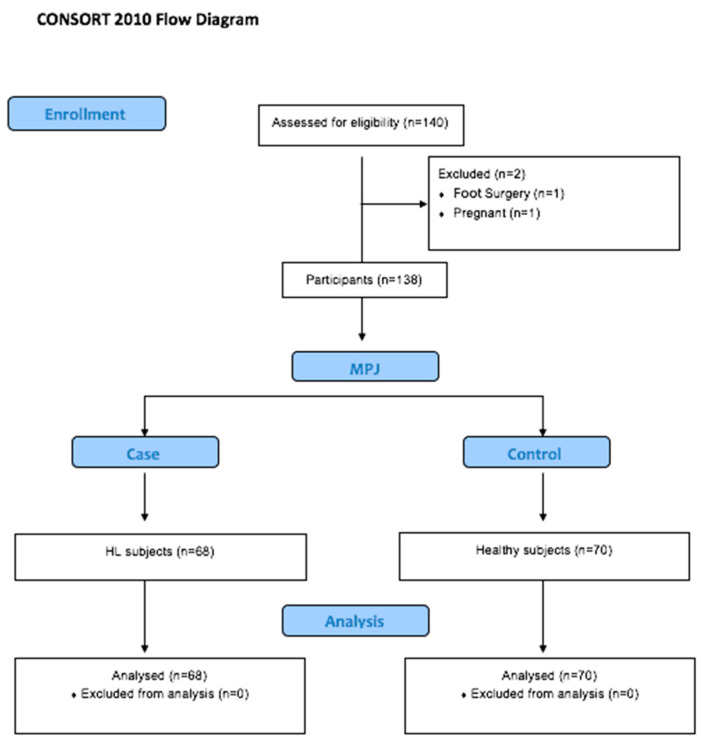
CONSORT flow diagram.

A comprehensive sample size of *n* = 138 individuals was enlisted from the Podiatry Clinical Area at the University of Malaga. This encompassed *n* = 68 participants with hallux limitation (forming the case group) and *n* = 70 individuals who were in good health (comprising the control group; Figure 1).

The inclusion criteria were (for the case group): subjects of 18–65 years of age with MPJ dorsal ROM < 60° (this was considered the minimum range of dorsal movement needed for normal propulsion movement in the gait cycle) [8,9]. Exclusion criteria for both groups were: previous diagnosis of degenerative diseases in the foot or lower limb or previous foot surgery. The inclusion criteria for the control group were being aged between 18 and 65 years and have a normal MPJ dorsal range (more than 60°).

The Seville Experimental Ethics Committee evaluated this study (CD 0966-N-20). The study was conducted taking into account the guidelines of the Declaration of Helsinki and was registered according to the STROBE declaration guidelines.

All subjects voluntarily agreed to participate in the investigation, obtaining informed consent for all biomechanical analyses. The evaluation of the feet was performed by a biomechanics-trained podiatrist (F.J.C-S).

All participants were placed in a supine position with the knee fully extended, the subtalar joint neutral, and the ankle 90° to perform biomechanical measures. During the measurement of ankle dorsiflexion, the Silfverskiöld test with two hands was performed to neutralize the hindfoot, midfoot, and forefoot, so that the dorsiflexion motion is only through the ankle joint [18]. The normal minimum range of dorsiflexion of the ankle joint was considered 10° with the knee fully extended and 20° with the knee flexed.

**Figure 2:**
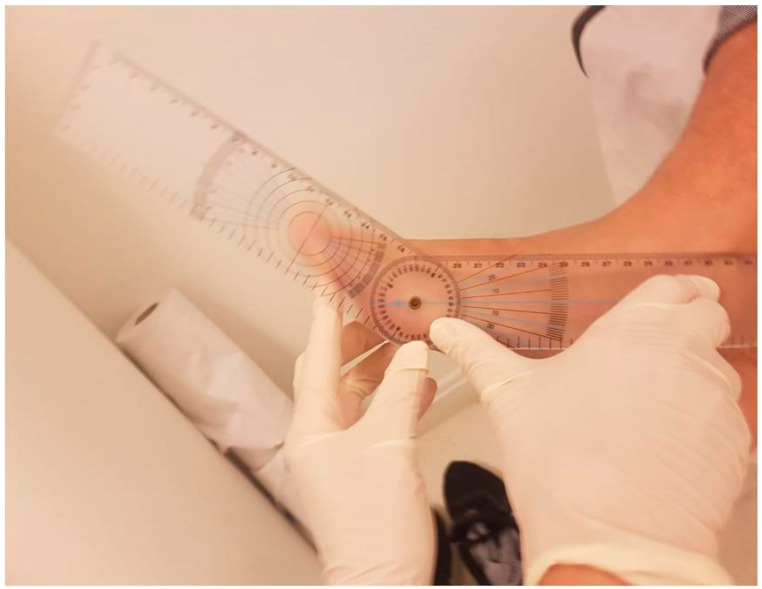
Passive MPJ dorsal ROM.

For the joint exploration of the first MPJ, the patient was placed in a supine position on the table. Measurements necessary for the study were made with the knee extended and the foot in a relaxed position. The instrument used to quantify the passive mobility range of the first MPJ was a two-branch plastic goniometer. It is a measuring instrument made up of two mobile branches that assesses the movement around a centre of rotation, the point of union between the two. In this relaxed position with the ankle at 20–30° of plantar flexion, the centre of the goniometer was placed in the centre of the metatarsal head. The proximal branch was positioned parallel to the bisection of the first ray and was attached to the foot with one hand. The distal or mobile branch was placed parallel to the bisection of the proximal phalanx and was attached to the finger with the other hand. In this position, the first of our measurements was obtained and corresponded to the physiologically relaxed position presented by the first MPJ (Figure 2).

The validated foot posture index (FPI) system was used to identify the type and measure the structural position of the evaluated feet (pronated, supinated, or neutral) [19].

According to this standard protocol, gait analysis was used with the one-metre system, which is the most recommended by the OptoGait System (Version 1.6.4.0, Microgate, Bolzano, Italy). To minimise gait cycle asymmetries, the participants performed three 30 s exercises on the treadmill with natural walk trials in a single measurement session. In both groups, the selected speed was 4 km/h. The protocol standard was performed three times, and the third measure was considered valid.

The spatiotemporal values between the right and left feet were compared, and when the values differed considerably, they were considered abnormal. The parameters analysed were: stride length in centimetres for each foot, stride length duration in seconds, ground contact time of the right and left foot in seconds, and data on the gait cycle duration in seconds and the gait cadence between subjects in steps per minute (SPM). The research protocol was conducted as described in other studies [16,17,20].

The sample size was computed to achieve a power of 0.90, with an alpha error of 0.05, and a size effect of 0.5 (testing method: T test, G* Power 3.0.10, Franz Faul, Kiel University, Kiel, Germany). A total of 65 participants were deemed necessary for each group. Initially, a total of 130 individuals were enlisted. Ultimately, two participants did not meet the predetermined research inclusion criteria.

An initial exploratory descriptive analysis was undertaken: encompassing qualitative and quantitative variables, assessment of variable distribution (Kolmogorov–Smirnov test), and group comparisons (bivariate analysis of qualitative variables using the chi-squared test and quantitative variables through Student’s *t* test for independent groups, prior to assessing normality). The Mann–Whitney U test was employed when appropriate. In instances of comparing two quantitative variables, the Pearson test was applied for parametric samples, and a Spearman test for variables exhibiting nonparametric behaviour. All data were reported as mean ± standard deviation (SD), while median and interquartile ranges were employed for nonparametric data. All analyses were executed using SPSS^®^ version 24.0. A significance threshold of *p* < 0.05 was adopted. An intention-to-treat analysis was conducted.

## 3. Results

### Description of the Total Sample and by Groups

The final sample size was a total of 138 participants. The results show a larger sample of women than of men (46 men and 92 females). The average age was 25.86 ± 10.95 years (range of 18–61 years) and the body mass index (BMI) was 22.20 ± 5.12 (normal weight). An analysis was conducted for the variables age, sex, BMI, dorsal MPJ range, and FPI for each foot of the total sample, for both groups. Data are shown below (Table 1).

Table 1 shows a significant *p*-value in the baseline value with respect to the ROM of the MPJ, the FPI, and the ankle ROM in both feet between the case and control groups. The sample was homogeneous with respect to body mass index (BMI) and left foot FPI. The next analysis shows the descriptive results for the gait parameters between both groups after using the OptoGait sensor. These outcomes are outlined in Table 2.

The findings indicate statistically significant outcomes for various variables between the two groups: right and left stride length, right and left stride time measured in seconds, gait cycle, and cadence (in all instances, *p* < 0.05). Moreover, notable distinctions were observed in step length, stride length in centimetres, and loading time. The hallux limitation group exhibited a decrease in gait cadence, disparities in load response, and variations in passage time.

Upon comparing the cases with controls, the results demonstrate significance in stride length for both the right and left feet (*p* = 0.001 and *p* = 0.001, respectively). Significant differences were also identified in the ground contact time for both the right and left feet in seconds (*p* < 0.001 in both cases). Additionally, the gait cycle and cadence displayed statistical significance between the groups (*p* = 0.001 and *p* = 0.001).

## 4. Discussion

A normal gait cycle is not easily defined. There are studies that examined parameters in healthy subjects and a preliminary framework of normative reference data has been provided, which provides insights into the influence of age on spatiotemporal parameters [21,22]. However, the spatiotemporal parameters of middle-aged adults with musculoskeletal disorders, such as HL, should be investigated more thoroughly.

The OptoGait optical data sensor is a system that provides asymmetric gait evaluation. The use of devices in paired subjects has been useful in detecting gait cycle alterations between both groups, and studies with paired subjects have been useful in detecting gait cycle alterations that can be related to foot disorders. The results obtained with the OptoGait test system can be used to correct for asymmetry in gait parameters by using custom foot orthoses to normalize gait and improve foot biomechanics [23,24,25,26].

The aim of this study was to assess whether there are changes in the spatiotemporal characteristics of walking, as measured by the OptoGait system, among individuals with HL when compared to a well-matched group of healthy controls. As far as our understanding goes, this research marks the initial instance of presenting novel proof regarding gait cycle deviations captured through the OptoGait optical sensor system in a cohort of individuals affected by first toe pathology, specifically HL, in comparison to their healthy counterparts.

Drawing from the findings derived in this study, noteworthy alterations in the spatiotemporal aspects of the walking cycle, as measured by an OptoGait optical sensor, were evident between the group of individuals with the condition under investigation and the control group. These differences manifested themselves in parameters such as step length, stride length for both the right and left foot, and loading time, all of which exhibited statistical significance (*p*-value < 0.05), as outlined in Table 2.

These results are consistent with those of previous studies. In the present study, the feet of the individuals in the study group showed less ankle dorsiflexion, which could lead to their greater dynamic pronation. The spatiotemporal parameters obtained in our study were very similar to those observed by Requelo et al. in a group of subjects with pronated feet [20]. Due to the novelty of this topic, a discussion is difficult due to the impossibility of comparing our results with those of other studies using the OptoGait system.

Some case–control studies have shown that the presence of HL, structural or functional, causes the maximum plantar pressure under the hallux to accumulate more significantly and at a faster rate than in the first metatarsal head, with a consequent increase in pressure in the rest of the forefoot [1,3]. Dananberg affirmed that the limitation in hallux movement in the propulsive phase of gait, when repeated thousands of times daily, does not only alter foot and postural biomechanics, but also causes and perpetuates many chronic postural alignments, including lower back pain [2].

Some studies concluded that the consequences of this HL dysfunction can affect all age groups and manifest itself in the form of lower back pain, impingement, sprain, joint incongruity or tendon overload, and fibro-osseous injuries, showing a gait pattern with increased stress applied to the bones and joints and, subsequently, impaired balance [27].

Wearable technology is a practical way to detect deterioration in gait balance and physical activity that can be associated with foot problems. Several studies have used different gait analysis systems and wearable sensors with the aim of accurately measuring body motion and providing interactive feedback to support motor learning in subjects with and without biomechanical alterations of the foot. As in the results of our study, the results showed that subjects with foot alterations exhibited a different stride length compared to healthy people [20,28].

In conjunction with our study, other authors using different analysis systems have observed that patients with HL have gait disturbances. Canseco et al. observed a prolonged stance phase in patients with HL that showed significant alterations in gait patterns compared to controls on various planes in all segments (hallux, forefoot, hindfoot, and tibia) of the foot and ankle, particularly in the hallux and forefoot range of motion [29]. Furthermore, the analysis revealed significantly longer stance durations, with notable reductions in walking speed and stride length and shorter stance duration [26,29]. In the same way, other studies added that, as the angle of dorsiflexion of the first ray decreases in the structural HL, the angle of progression of the gait decreases [30].

In the same way, recent studies have indicated that individuals with fewer passive non-weight-bearing first MPJ maximum dorsiflexion exhibit less dynamic first MPJ dorsiflexion, less plantarflexion of the ankle joint, and less total excursion of the ankle joint during level walking and, in turn, present an altered gait pattern, characterised by a reduced stride length and a shorter stance duration [31]. Changes observed in gait after hallux metatarsophalangeal arthrodesis using a three-dimensional optoelectronic system have shown a decrease in the maximal dorsiflexion of the ankle joint and a significant decrease in propulsion force in the anteroposterior and vertical planes [30].

Hence, the findings of these studies corroborate our outcomes; HL changes the dynamic characteristics of walking and thereby impacts its optimal progression. The spatiotemporal parameters of the gait cycle were associated with significant modifications in the group of HL subjects compared to the group with normal feet in terms of the stride length in centimetres of the right and left foot and loading time.

In the present study, only spatiotemporal gait patterns were evaluated. In future studies, other elements could be evaluated to corroborate that HL patients may have a higher risk of falls than healthy patients. This can be interesting from the point of view of prevention, especially in the elderly, through the early compensation of the foot, such as the use of plantar supports, which allow for a more stable and balanced gait. Observational studies have shown that foot problems are associated with frailty level and decreased motor performance [32].

Therefore, improved gait in patients with HL can successfully improve quality of life or even avoid the risk of falling for improved gait, especially in older adults with functional limitations in biomechanics and gait imbalances. Utilizing wearable technology offers a pragmatic means of identifying declines in walking patterns, balance, and physical engagement that could potentially correlate with issues related to the feet. The regular evaluation and addressing of foot concerns have the potential to facilitate prompt interventions, aiding in the preservation of motor abilities and the mitigation of apprehensions regarding falls among the elderly population.

Gait disturbances and loss of balance can cause falls especially in the elderly population, so it is important to avoid such alterations. If we can detect foot and walking issues at an early stage, we have the opportunity to introduce preventative measures aimed at restoring the natural biomechanics of walking. Patients with musculoskeletal disorders, such as HL, can suffer from falls to a greater extent than healthy patients.

In our study, only spatiotemporal gait patterns were evaluated. In a future study, a questionnaire on the risk of falls could be evaluated in these patients to corroborate that patients with HL suffer a higher risk of falls than healthy patients. This holds potential interest in terms of prevention, especially in the risk of falls in the elderly by the early compensation of the foot. For instance, utilizing insoles that provide arch support can facilitate a gait that is both steadier and more harmonized. These connections could play a pivotal role in shaping novel intervention approaches aimed at preventing issues in individuals with hallux limitations.

As a limitation of the study, we found that the sample of women was larger than that of men (46 men and 92 women). In a future study, it would be necessary to consider that the sample of men and women was totally balanced so that it would be representative and comparable.

## 5. Conclusions

The presence of a structural HL can alter the normal gait patterns of the gait cycle. This study used optical systems, such as OptoGait sensors, to detect the spatiotemporal alterations of gait. We believe that the early detection of these alterations may allow the establishment of therapeutic strategies to improve quality of life and prevent complications associated with the future, especially in the vulnerable population.

## Figures and Tables

**Table 1:** Values of the entire sample of demographic variables for both groups. The ROM of the MPJ and the ankle and foot posture index for the right and left feet in degrees (average ± standard deviation) are shown.

Sample *n* = 138		Group		
		Case *n* = 68	Control *n* = 70	*p*-Value
Gender Female	92 (66.7%)	44 (64.70%)	48 (68.6%)	*p* = 0.630 ^a^
BMI (kg/m^2^)	22.20 (5.12)	21.90 (5.20)	22.80 (3.50)	*p* = 0.436 ^b^
Age	25.86 (10.95)	23.03 (8.86)	28.60 (12.08)	*p* = 0.001 ^b^
ROM MPJ right foot (degrees)	67.00 (38.00)	52.00 (10.00)	90.00 (14.50)	*p* = 0.001 ^b^
ROM MPJ left foot (degrees)	75.00 (30.00)	60.00 (11.00)	90.00 (24.00)	*p* = 0.001 ^b^
FPI right foot	6.00 (3.00)	7.00 (1.00)	6.00 (3.00)	*p* = 0.012 ^a^
FPI left foot	5.00 (1.00)	5.00 (3.00)	6.00 (3.00)	*p* = 0.590 ^b^
ROM right ankle (degrees)	11.50 (12.00)	9.00 (7.00)	15.00 (9.00)	*p* = 0.001 ^b^
ROM left ankle (degrees)	11.00 (12.00)	8.00 (4.00)	15.00 (8.00)	*p* = 0.001 ^b^

^a^ Chi-squared test. ^b^ Mann–Whitney U test. Values are presented as median (interquartile ranges). BMI: body mass index. ROM: range of motion. MPJ: metatarsophalangeal joint.

**Table 2:** Descriptive statistical analysis after the OptoGait sensor gait analysis between the control and case groups.

Sample *N* = 138		Group		
		Case *n* = 68	Control *n* = 70	*p*-Value
Right foot stride length (cm)	61.20 (6.90)	64.50 (5.40)	59.20 (6.02)	*p* = 0.001 *
Left foot stride length (cm)	61.15 (6,60)	62.95 (5.20)	58.20 (6.58)	*p* = 0.001 *
Ground contact time (loading time)				
right foot sec Step (%)	0.55 (0.05) 70.90 (6.63)	0.57 (0.04) 69.30 (3.30)	0.53 (0.06) 69.00 (3.03)	*p* = 0.041 * p = 0.460
Ground contact time (loading time)				
left foot sec Step (%) Gait cycle sec Gait cadence	0.55 (0.05) 70.60 (6.00) 1.10 (0.10) 108.50 (8.83)	0.57 (0.04) 69.80 (2.40) 1.14 (0.09) 106.40 (13.20)	0.53 (0.06) 68.65 (2.90) 1.07 (0.05) 112.20 (3.70)	*p* = 0.003 * p = 0.081 *p* = 0.001 * p = 0.001 *
Passage time difference	0.32 (3.75)	0.30 (4.25)	0.45 (3.52)	*p* = 0.040 *
Step length difference (cm)	0.45 (6.00)	0.40 (8.25)	0.50 (5.35)	*p* = 0.255
Total stride (cm)	123.00 (11.50)	127.80 (8.75)	118.40 (12.03)	*p* = 0.001 *
Difference load response	−1.40 (6.32)	0.30 (4.75)	2.20 (9.43)	*p* = 0.027 *

A Mann–Whitney U Test was used. Significance set at *p* < 0.05 *.

## Data Availability

Not applicable.
